# The Child With a Disability: Parental Acceptance, Management and Coping

**DOI:** 10.1100/tsw.2007.265

**Published:** 2007-11-12

**Authors:** Isack Kandel, Joav Merrick

**Affiliations:** ^1^ Faculty of Social Sciences, Department of Behavioral Sciences, Ariel University Center of Samaria, Ariel, Israel; ^2^ National Institute of Child Health and Human Development, Jerusalem, Israel; ^3^ Office of the Medical Director, Division for Mental Retardation, Ministry of Social Affairs, Jerusalem, Israel; ^4^ Kentucky Children's Hospital, University of Kentucky, Lexington, USA

**Keywords:** Mental retardation, intellectual disability, physical disability, developmental disability, process of acceptance, environmental variables

## Abstract

Research indicates that family reaction to the birth of a disabled child changes according to the type of disability and the child's diagnostic category. The differences are probably an indirect consequence of anticipated or actual reactions by those surrounding the disabled child and the family, in addition to parental reactions. Many researchers have recently mentioned the positive coping and functioning of many families with developmentally disabled children. In the past there was a tendency to emphasize issues of illness and pressures, spousal strain and maladjustment within the family, while presently they are replaced with questions concerning positive adjustment, satisfaction, acceptance, and spousal harmony. Rather than perceiving the family as a helpless victim, it is perceived as a unit that adapts by a process of structuring. Professionals must acknowledge the importance of the family, this change towards a positive attitude towards disability and that the controls decisions concerning the disabled child and the family.

## INTRODUCTION

Families are dynamic functioning units repeatedly propelled from situations of stability and balance to those of development and change. Individuals and their families evolve within a single system, constantly striving for balance. The birth of a child with developmental disabilities creates a severe breach of this balance and the family undergoes a difficult existential experience[1,2,3,4].

Parents are the central and most important link in the care, education, and supervision of persons with intellectual disability (ID). Despite this major role, the literature tends to minimize their significance. Even in Israel, despite the great importance of family, the role of parents is rarely discussed. Professional literature dealing with parents' patterns of coping with raising a handicapped/disabled child describes a wide spectrum of patterns, ranging from reactions of mourning and crisis to those of acceptance[2,3,4]. It is very important to examine parents as coping people and the developmentally disabled as children, adolescents, and adults with special needs.

Prior research has tended to examine the coping of dysfunctional families, while families coping successfully with crises were not sufficiently investigated. Recently there has been a revolutionary change in the professional perception of families of developmentally disabled people. The monolithic concept of families coping with raising a developmentally disabled child as inevitably located in a situation of crisis, stress and pathology has evolved towards multi-dimensional recognition of many varied manners of coping. This concept acknowledges the need to investigate and understand the causes of such varied coping patterns. Understanding the varied patterns can contribute significantly to theories dealing with stress, coping, and adjustment, and lead to the development of more efficient methods of intervention in working with these families. The change in concept originates from a number of developments:

Where once the focus was only on the disabled children, the last two decades have seen an increased interest in the entire family. This is manifested both in the large number of studies on the subject and in the development of additional services geared for families. In the United States this change is also expressed by legislation acknowledging the role of families as the most important conduit for the development and service of disabled children. The movement towards noninstitutionalization and normalization, which began in the 1960s in Scandinavia and continues today, led to a situation in which more children with disabilities live at home, resulting in acknowledgement of the need to recognize and understand their families in order to construct an appropriate system of services. In the theoretical field as well, psychological theories dealing with stress, pressure and coping, yielded more complex models of coping and adaptation.

These models emphasize cognitive and environmental components. Changes in professional approaches to the adoption of children with developmental disabilities have also led to a significant increase in the number of adopted children, a fact that prepared the way for research on families, who have chosen to raise a developmentally disabled child. People coping with this role with outstanding success have enabled identification of other important components that affect coping, such as: religiosity, cognitive perceptions, values, etc.

In addition, longitudinal research following parental reactions from the moment a disabled child is born and examining their coping in various points of time throughout life is conspicuously absent. It is currently accepted that coping patterns change over the different life stages of the developmentally disabled and their families. The literature indicates the adaptive and positive functioning of families of young developmentally disabled children, but we know much less about families comprised of elderly parents of adult children with developmental disabilities[5,6]. Examination of stressful situations at various life stages found that developmentally disabled adolescents form the most difficult period of stress. Parents' aging and their prolonged non-normative parental role leads to the development of new problems requiring attention. Scientific findings in this area are not compatible. Some researchers indicate diminishing support and increasing stress, while other studies indicate an opportunity for psychological growth and challenging coping processes. The discrepancy emphasizes the importance of following these families throughout their life span and identifying turning points requiring intervention.

Families' coping and their capacity to withstand the significant challenges they face determine disabled children’s functioning and social integration to a great degree. Families' capacity to prepare their children for anticipated confrontations in the real world is a major determinant of these children’s success as adults. This indicates the great significance of understanding family interpersonal relationships, focusing on the spousal relationship, other siblings, and the disabled child. Identifying the special needs of children and people with developmental disabilities and determining the correct policies to answer these needs are essential for successful rehabilitation and integration of handicapped people in society, while providing them with maximum opportunities to realize their potential and maintain their quality of life. Israel is exposed to developments in the field of developmental disabilities in Western countries. Professionals, disabled people and their families are aware of the various theoretical approaches and life style models, however these are not always suitable for Israeli reality. Accordingly, Israeli-based research is very important, as it attempts to take into consideration unique local conditions.

## The Birth Of A Child With Disabilities

The birth of a child is a sign of faith and hope, prompting expectations of continuity and perpetuation. The mere existence of the newborn, the baby's traits and appearance, are usually a source of pride. All parents have plans and expectations for their children, often imagining future scenarios and the child's advances (first steps, nursery school, elementary school, military service, college, etc…). The child's success is perceived as the parents' achievement. When a handicapped child is born, all expectations and hopes are dashed. It is difficult to perceive this child as continuing the parents. The child is no source of pride – rather a source of great disappointment. Parents of handicapped children undergo a difficult and painful process involving a revision of their views and expectations. They are obliged to adapt to the knowledge that all their hopes and plans for the future must change[2,7].

There is no tested way to help parents avoid the shock and pain that accompany exposure of their child's handicap. This is precisely why it is necessary to try and prevent any exacerbation and aggravation of their suffering. Physicians are usually responsible for conveying the news, however they do not always have the appropriate training in order to help the parents at this difficult moment of disclosure or the appropriate skills of communication and support. In addition, most physicians do not perceive this task as part of their responsibilities. Physicians use various tactics, when required to tell parents that their child has been diagnosed with a "defect". Some ignore, avoid, and delay the moment of disclosure, try to falsely allay true concerns, or hide behind a wall of scientific objectivity and describe the problem in medical terms, without explaining and detailing consequences for daily life, care, and prognosis. Some physicians choose to tell the father and entrust him with the responsibility of telling the mother. Sometimes all hospital medical and nursing staff form a joint bond of silence, in an attempt to circumvent and ignore the mother. Even when physicians assume responsibility for revealing the truth, they may encounter many difficulties, as a result of the parents' immediate reactions: unreceptiveness and listlessness, decline and denial, and even aggression aimed at the physician. These are possible defensive reactions of the parents, who are still unable to cope with the stressful situation forced on them. The parents associate the physician, who relates the harsh tidings with the disaster that has befallen them and therefore the physician is sometimes the direct target of their anger and hostility. Parents' helplessness burdens the physician. Distressed parents' ambivalent attitude to the medical establishment might increase, if they encounter reactions of avoidance. A frequent problem related to the parent-doctor relationship occurs when the mother initially discovers the child's problem and turns to the physician for confirmation or disconfirmation. In many cases physicians are reluctant to accept parental concerns and tend to reject them out of hand, as unreliable information rife with emotional involvement. The concerned parents succeed in breaching the wall of professional supremacy only after an exhausting pursuit of various physicians. Physicians encounter a number of major dilemmas:

*How should the diagnosis be presented to the parents with minimal damage?* Should the disclosure be postponed or hastened? Some physicians hope that the later they tell parents about their child's disability, the smaller the risk that the parents will reject or abandon the child, however this premise has no basis in fact. In many cases infants were left in the hospital or placed in institutions even though the defect was only disclosed after a period ranging from one week to one year…

*Should both parents be told together or each separately?* This question must be considered anew in each case, however an important consideration is the possibility that the burden on the parent who knows and is forced to conceal the knowledge from the other spouse might be detrimental to this parent and to the spousal relations precisely when there is most need of mutual support.

If the medical staff is equipped with thorough understanding of reactions in stressful situations, this might provide them with suitable skills and approaches facilitating optimal care at this stage, the first stage of the lengthy and difficult battle to which the family is being propelled. It is necessary to understand the family's extraordinary need for support combining authority and reliability, as their new situation as parents of a disabled child makes them dependent on the services of physicians and other caregivers, for better or for worse. From the moment the parents become parents of a disabled child they also become vulnerable and they require supervision, care, and support[2].

After receiving information of the child's handicap, parents enter a situation with the potential for crisis. Various studies have proven that the birth of a special needs child might breach intra-family balance[2]. The frustration and the failure experienced as a result of the birth of a disabled child, a child who does not conform to expectations, as well as coping with increasing special needs, create previously nonexistent responsibilities. Several studies have emphasized the functional crisis experienced by mothers and fathers of children with ID, stating that this crisis is accompanied by severe disappointment, psychological stress, a strong feeling of loss, and reduced self-esteem[2].

An endless list of possible reactions of parents of disabled children, e.g.: anger, embarrassment, concern, anxiety, denial, confusion, rejection, ambivalence, bitterness, idleness, over-protection, shame, self-pity, shock, deep pain, sorrow, depression, hostility, mourning, wish to kill or suicide attempts[8] and parental reactions can be divided three types: The first type is called "the crisis of change" and it stems from the occurrence of an unexpected change in the individual life and self-perception. This is not a reaction to the disability per se, rather to the sudden change in life circumstances. The second type of crisis is connected to the transformation of individual personal values as a result of the specific crisis. Most people have been educated according to an ethical system that stresses individual personal abilities and achievements. The birth of a disabled child requires parents to love a significant figure – their child, who is deprived of the ability to grant a feeling of achievement. The result is bivalent feelings toward the child. A third type of crisis is called "the crisis of reality" and it stems from the harsh objective conditions formed by the need to raise a handicapped child: financial difficulties, limitation of the parents' free time, and the great deal of time that parents are required to devote to their child.

## Inquiry and Acceptance

The above provided a review of families of disabled children, the various crises experienced by the family over time, such as: the crisis of change, the crisis of personal values, and the crisis of reality. Families of disabled children typically experience all three crises, however these are not necessarily equal. Some crises will last longer than others. If the family succeeds in rising to the challenge and enduring these crises it reaches the stage of acceptance, i.e. – acceptance of the child, as will be explained forthwith. In the process of acceptance the family is capable of beginning to care for itself and for the disabled child according to a professional plan, to solve the conflict, and to accept the child despite the limitations. The professional literature does not stress positive manners of coping, as it tends to describe negative aspects of the coping process. There are undoubtedly healthy parents who react appropriately to a situation in which they must live with a disabled child. These are parents who consciously adapt to their child. In order to reach the stage of acceptance and to facilitate the child's advancement and rehabilitation, the child's actual situation must be accepted and the problem must be acknowledged. In order to reach a balance between excessive expectations, which end in disappointment, and "giving up", it is necessary to create a fundamental change in approach. For this purpose it is very important to have basic faith in the child's potential, whatever the disability. At this stage the parents search for solutions to their problem and ways to help their child advance. They learn to appreciate their inner strength to deal with the affliction and consider alternative solutions. They learn to understand the essence of the affliction and the limits within which the child may develop towards independence. They learn to use existing community services and benefit from them. Parents usually utilize the assistance of professionals and therapists for this purpose.

Families that accept their handicapped child are defined as being in a state of balance between recognition of the child's limitations and seeking to compensate for these limitations, while also avoiding burdening of intra-family communication processes. Four characteristics of the process of acceptance have been identified. The four characteristics of the process of acceptance:

- Suitable parental perception of the disabled child's skills and capabilities with appreciation of the child's weaknesses and limitations.
- A realistic view of the child, with appreciation of the complications created in the family; the parent is not overwhelmed by feelings of self-pity and guilt.
- The parent engages in a logical search for possible services and does not seek a "magical solution".
- The accepting parent is able to provide love to the disabled child, with no feelings of rejection or over-protection; in addition, the parent is careful that this is not at the expense of attention provided to other family members[9,10].

In the 1990s various researchers[11] related to family resilience in positive terms, examining the family's strength and ability to resume its previous condition – the ability to rehabilitate itself or to overcome the issue of disability. The use of new terms such as resilience, survival, and coping was based on the assumption that the family can resume its basic pre-event functioning and emphasizes the family's robust forces.

Aaron Antonovsky (1923-1994)[12-19] called for consideration of "healthy" aspects and of power sources within the personal and familial system (salutogenesis) rather than on aspects indicating a "pathogenic orientation", such as illness, feeling of helplessness or "me as victim" self-image (pathologenesis) , i.e. on "salutary factors" rather than risk factors. Within the family, for example, it is necessary to focus not only on the special needs child or the family's hardships and difficulties, rather also on positive familial resources that contribute to proper functioning, family members' mutual support, relationships, the "space" of each of the family members, and the emotional atmosphere. Rather than identifying an individual with an illness, disability, or any other limitation, he preferred to examine how it is possible to help this individual or the entire family system to overcome the difficulties instead of ignoring them.

The term "resilience" denotes the ability to function in crisis situations, to demonstrate resourcefulness, the ability of survival and of adaptation. The family's resilience is related to the relationship between family members, to their cooperation, to their ability to solve problems within the family and make use of various sources of support, and also to a coherent perception of reality, intellectual flexibility, and the ability to use existing resources in order to overcome a given situation. The individual's personal traits, as well as family resources and sources of social support, may help family members gradually resume normal functioning. Some see resilience as an acquired skill that develops following recurring experiences, while others think that it is an innate quality, a type of biological inflexibility and ability to withstand pressures with which a person is born. This ability is also influenced by the degree of support that a child receives from the family and by the functioning of the family's adults.

## DISCUSSION

Families' coping patterns depends on a wide variety of factors, like personality, support system, education, financial situation, spousal relations, family cohesion, and the level of the child's handicap. In order to examine the reason for differences between families, who seemed to cope well and families who do not, the correlation between a variety of variables was examined, including the child's traits, family structure, and family members' feeling of well-being[11].

A qualitative study examining the daily routine of families with a developmentally disabled child identified a number of conspicuous characteristics of parents who have a high level of coping[20]. One of these characteristics regards perceptions and thoughts regarding the situation, and the ability to reevaluate the situation and to reformulate initial negative thoughts is a significant component of efficient coping in the case of parents raising a developmentally disabled child. More positive thoughts, which facilitate better coping, center on acceptance of the disabled child in the present state, determination to succeed, and finding a goal or meaning for the situation. Families who cope well find a proper balance with roles and responsibilities towards other children, spouses, work, and personal needs, while also devoting time and resources to physical and emotional health and nurturing spousal relations, in a manner that provide a response both to the child's needs and to the needs of other family members. Efficient coping was also found to be related to efficient seeking and use of resources: gathering information, cooperation with professionals, forming contact with other parents, and integration in support groups.

In any case, since the birth of a disabled child occurs within a very clear context of a certain family structure, at a certain developmental stage, while concurrently coping with other challenges, it can be assumed that the more proficient and varied the family's resources before the birth of the disabled child – the easier it will be to adapt and cope efficiently with the new situation.

## Personality Variables

1. Coping style: The literature dealing with stressful situations indicates that there is a connection between style of coping - and psychological and physical symptoms. Problem-focused coping is described as a normally efficient strategy, particularly when the situation is under control. Emotion-focused coping strategies, which according to some of the researchers include searching for emotional support, have sometimes been found to be more efficient in situations perceived as uncontrollable. In contrast to such strategies, avoidance, i.e. behavioral and/or mental dissociation from the stressful situation, was found to be less effective[20].

2. Personal hardiness: According to Kobasa[21], personal hardiness expresses an array of personality traits that are correlated with a person's resilience when faced with stressful life events. This array includes commitment, control, and challenge. A correlation was found between commitment, expressing a tendency to involvement, and the ability to find meaning in other people. Regarding control, when an individual is able to act in control – this leads to more active and tenacious coping with stressful situations. Anticipating change and perceiving it as a source of development and not as a threat to self-confidence due to consequent instability express challenge. The major claim is that commitment, feeling of control, and challenge, all assist people who encounter stressful situations. The dimensions of control and challenge represent a cognitive position, since assessing a situation as controllable or challenging (or both) increases the individual's motivation to cope and thus improve the ability to adapt. This variable is also related to the individual's hopes, as hope increases motivation to cope due to a cognitive position in which the situation is evaluated as less threatening. Rimmerman[22] examined if focused control and social support alleviate stress and consequently affect applications for institutional placement by parents of adolescents with severe ID. Parents who applied for institutional placement of their children were found to have an external focus of control. This was manifested in feelings of helplessness and passivity. In contrast, parents with an internal focus of control, manifested by animation and belief in their ability to cope with difficulties, had less of a tendency to perceive institutional placement as the solution to their difficulties.

A qualitative study[11] examined the personality traits characteristic of efficiently coping parents and six main qualities were identified: flexibility, patience, perseverance or determination, positive and hopeful approach, sense of humor, and willingness to accept help. Findings regarding the control variable were less uniform: While efficient coping was related to parents' feeling of control of the situation (decision making etc.) parents paradoxically stated that an important component of efficient life management was the ability to live with a certain lack of control over daily life and over the future.

This may lead to the conclusion that improving and reinforcing a family's range of strengths, including coping strategies, might provide wide support to families with developmentally disabled children. One of the goals of early family-focused intervention is the identification of strengths and abilities that exist in the family in order to benefit from intervention that is based on existing patterns. Naturally, using the family's strengths as building blocks leads to empowerment of the family and its self-perception as capable of coping with the various needs of its members. Some studies show those parents'

efforts to cope by seeking social support that would provide for emotional and informational needs anticipate family coping forces. In contrast, self-accusation, distancing, and avoidance, were found to be negatively correlated to the family's strengths.

## Parental and Family Variables

A systemic approach to families may illuminate the relationship between the family system and its manner of coping with a developmentally delayed child. In one case[11] families were divided into three categories: families with clear limits, families emphasizing over-cohesion, and dissociated families. Families with developmentally delayed children usually belonged to the model of over-cohesion or dissociation/distance. It can be assumed that as a result of the pressures involved in raising a disabled child the family structure takes a turn for the worse, towards less efficient family functioning.

The double ABCX model of family coping and adaptation emphasizes family competence in regard to the manner in which it adapts to or copes with demands over time[23,24]. This model is a theoretical framework for assessing family coping, where coping is a function of stressors, demands, and interaction between resources, perceptions, and behaviors. The model assumes that the import of an event as perceived by the family holds much significance for its process of adaptation and that different families relate to stressful experiences in different ways, according to the family's resilience in its attempt to reach a state of balance. The family may encounter a variety of demands, including daily demands, continuous strain resulting from initial stressors, as well as dramatic crises. The family's functioning stems from its skills and internal hardiness, and in particular its tendency to flexibility when necessary and the ability to empower others as part of a process of development and coping. In time the family develops a family scheme for coping, which will guide it in crisis situations. Rehabilitation professionals were greatly influenced by this model of coping in stressful situations when diagnosing and evaluating the coping strengths of families of children with developmental disabilities[24].

A more innovative model of family adaptation, termed AAR (Family Adjustment and Adaptation Model), attributes much significance to the family's perceptions, while distinguishing between situational and global meanings. Situational meanings are unique meanings developed by the family in connection with capabilities and demands for a specific crisis situation, while global meanings are normally unchanging stable and general perceptions and beliefs. Families succeed in adapting by reaching a balance between these capabilities. The model states that different demands stem from meanings attributed by family members. Thus, if the mother has unrealistic expectations of the developmentally disabled child she might experience a great deal of stress in attempting to realize these expectations. Using cognitive strategies might reduce demands by lowering expectations, or empower abilities through evaluation of existing achievements[25].

A major issue that appears in the context of coping with raising a developmentally disabled child is the issue of role strain – a condition that reflects the manner in which parents evaluate their level of stress in regard to their role as parents. Parents might experience emotional burden, fatigue, and strain, due to many daily tasks, frustration, and burnout. These difficulties are accompanied by high social expectations of "good" parenting, which affect the parental image and self-esteem. Shtenger[26] lists parental traits that lead to parental role strain, including maternal image, feeling of competency, and also parental attachment. Situational traits may also affect coping, e.g. spousal relations, social support, and parents' health.

Coping with stressful situations as a function of interaction between the individual and the environment is emphasized in Pearlin's specialized model[25], which deals with stress stemming from two sources: life events and chronic hardships. According to this model, which examines the effect of the birth of a disabled child on spousal and family balance, parental traits are affected by an integrated system that includes variables from the world of the parent and the child and interactive variables that deal with mutual relations between the child and the parent and the degree to which the child's traits match those anticipated by the parents. For example parents might experience difficulty as a result of their guilt reactions to an unwanted child, and this guilt is accompanied by feelings of blame and embarrassment aroused by their antagonism towards the handicapped child[27].

As stated, much has been written about the family's ability to adapt and the parents' level of flexibility. This variable is related to the ability of parents raising a developmentally disabled child to form close relations and to interpret life events positively. Parents with a high level of flexibility also tend to have an internal control focus and to use efficient strategies to solve problems[28].

## Environmental Variables

The ecological approach, which focuses on individuals as perceived within their environment, examines variables such as employment, transportation, housework, and also factors related to safety (violent environment, racial discrimination, etc.) and the manner in which these variables shape daily routine. Cultural factors and values also serve as variables that influence coping, and routines maintained by the family in its coping with a developmentally disabled child are influenced by the family's values. Cultural goals and moral values regarding the definition of parenting and the development of joint living dictate the scripts for achieving these goals. For example, religious values are a means for interpreting and giving meaning to disability. In addition – living in a religious community often provides a support system facilitating daily coping. However family beliefs can also provide negative interpretations of the situation, such as perceiving the disability as a punishment. Religious faith is often perceived as beneficial for the family's adaptation since it provides a setting for cognitive and emotional interpretations and processing[28].

Weiss extensively discusses civilization's approach to conditions of anomaly[28]. In her opinion, it is necessary to identify the cultural orientation that determines who will be isolated and who will be embraced, who will remain at home and who will be estranged. In an attempt to explain phenomena such as abandonment of children in hospitals, neglect, and even abuse of developmentally disabled children, Weiss explains how parents' behavior may match cultural rules as well as the definition of children as parents' responsibility only as long as they do not endanger parents' social status and social life. The term "affinity" in this context embodies a process in which a child born disabled does not fulfill these criteria, and therefore there is no affinity between the child and the parent. Another term used by Weiss in reviewing a wide range of parental reactions is the threat inherent in the encounter with a state of anomaly, which is basically related to the prevalent need for cognitive order and to the clear limits defined between the concepts of "human" and "non-human". When people define their identity as the antithetical image of the "inferior" group, developmentally disabled children represent the risk of breached categories and cognitive ambiguity and serve as a significant threat to people's cognitive order and to their self-perception and identity. According to this explanation, transferring such children to the care of people who are not their parents' forms a favored solution as the self-definition of non-related caregivers is not subject to the same vulnerability.

However, we must be careful when attributing family coping to cultural, moral, or religious variables. Studies performed in Israel indicate that different approaches of ethnic and religious groups are often a function of interaction between a number of variables, of which only some are related to religious, cultural, or ethnic norms. For example, in Western society maternal relations with disabled children are mainly guided by guilt feelings and mothers strive to adjust their behavior to society's moral standards and values of society, however the dominant feeling in Western culture is that of shame. The fact that Westerners are occupied with avoiding experiences of shame and negative judgment explains the tendency to relate negatively to a person whose behavior or body traits are anomalous or different. Accordingly, the significance of cultural, ethnic, or national factors in regard to Israeli attitudes to developmentally disabled people is neither simple nor unequivocal, and no single variable pertaining to religion, culture, or ethnic group can fully explain the differences in attitude towards the disabled[26].

## Formal and Informal Support (Support Networks)

Formal and informal support systems (i.e. extended family, friends, neighbors, self-help group, etc.) have a very significant place in family coping with raising a developmentally disabled child, both when discovering the disability and later on. The earlier the intervention and the more support provided to the family, all family members will find it easier to function throughout their life cycle[29].

Social support is a human extrinsic coping resource. It helps alleviate or prevent pressure and answers the need for appreciation, affection, security, and belonging. Support networks help the family on a number of levels: relieve stressful situations within the family throughout the family's life span, provide emotional and material support including instrumental assistance, advice, problem solving and mediation, and particularly structure and preserve a social fabric that surrounds the family and the disabled child[26]. This network helps the family cope with situations of loneliness and alienation, reported by families with developmentally disabled children. Findings indicate that the larger the network, the greater its power to curb the influence of negative life events on moods and feelings of depression[30].

Weiss cites a possible relationship between cultural factors and environmental factors, particularly the existence of support networks[28]. She claims that a correlation was indeed found between people's religiosity and their willingness to accept their disabled child, however in her opinion intervening variables may explain this correlation. She states that the tendency of a religious person to avoid abandoning a disabled child does not stem only from a humane outlook, rather it is significantly influenced by the existence of dense social networks that facilitate care of the disabled child at home, not necessarily by the parents. A study held among ultra-orthodox Jewish families[29] found that parents utilized varied support networks, which included relationships with grandparents and conversations with family members as well as with education and health professionals. Laser[30] states that religious support is a factor that contributes to parents' acceptance of their handicapped child and to their adaptation.

The support system is perceived as a multi-dimensional system, which includes formal and informal components, in which the family and social system serve as a rich reserve of resources that can be used to fulfill needs and aspirations[26]. In contrast, the formal system is defined by Dunst, Trivette, and Deal[10], who refer to several areas of support, including functional, structural, and satisfaction. The functional index relates to the type of assistance and the nature and quality of the support, the structural – to the frequency of encounters, and satisfaction – to the efficiency of the support system (service accessibility and availability, degree of parental involvement in care, etc.). Parents tend to report more satisfaction when communication is sincere and open and when there is circumspect attention to the needs of the child and the family. They tend to cite dissatisfaction when they do not believe that the medical services are capable of helping. As the support system is considered very central for family coping and it has a direct bearing on the individual's personal well-being, it is discussed in most essays dealing with family coping. The defensive function of the natural social support system in family coping in time of crisis is widely accepted. The formal support network has a therapeutic function, and it includes healthcare, educational, and welfare services, which can help the family with its problems and challenges[10].

## CONCLUSIONS

Two conflicting concepts may be discerned in the literature dealing with the effect of raising a developmentally disabled child on the family throughout its life cycle. One concept stresses burnout, stating that continuous burnout reduces family resources. The other focuses on the family's ability to adapt and states that in time the family adapts and acquires tools and experience which may help daily care and bestow renewed, positive meaning, on this reality. However positive manners of coping are not stressed in the professional literature, which tends to describe negative aspects of the coping process. The assumption is that the family's reaction to the handicapped child will change according to the type of disability. For example, the family will react differently to a visible disability than to a non-visible disability (implying deformity), which is a hereditary developmental disability (in contrast to “incidental”). In the former case elements of self-blame are stressed. For example, families, according to the child’s diagnostic category, report different levels of mourning and strain. It may be assumed that some of the diversity is an indirect outcome, not only of parental reactions but also of anticipated or real environmental reactions to the disabled child and his family. Following this idea, I assumed that the acceptance process of parents of physically disabled children would be easier than the acceptance process experienced by parents of children with (medium and lower) mental retardation i.e. parents of children with physical disabilities would report a normal level of family functioning, a better family atmosphere and a normal spousal relationship, compared to families of children with mental retardation.

The professional literature tends to minimize the parents, the family and their coping with the developmentally disabled child. The professional literature dealing with parents’ coping patterns describes a range of patterns, from reactions of crisis and mourning to those of acceptance. Major emotions mentioned are guilt, denial, sorrow, tendency to over-protection and depression, and also patterns of parental acceptance. While in the past research tended to focus on dysfunctional family coping and to assume that family reactions to the birth of a disabled child are mainly uniform, today there is more attention to successful family coping with crises. The principle of normalization, the idea of integrating the disabled in the community, and even the developmental model, contributed to this transition in attitudes to family coping with the birth of a disabled child and to the development of an approach acknowledging a wide range of parental reactions.

Even if parents usually experience one or more of the crises discussed in the literature, including the crisis of change, the crisis of values, and the crisis of reality, most parents can adapt to reality and develop coping tools. Professional acknowledgement of these difficulties and family awareness of the normalcy of crisis reactions may help families currently in a state of imbalance.

Ref. 8 not noted in text.
